# Information transmission from NFkB signaling dynamics to gene expression

**DOI:** 10.1371/journal.pcbi.1008011

**Published:** 2020-08-14

**Authors:** Alok Maity, Roy Wollman

**Affiliations:** 1 Institute for Quantitative and Computational Biosciences, University of California, Los Angeles, California, United States of America; 2 Departments of Integrative Biology and Physiology and Chemistry and Biochemistry, University of California UCLA, California, United States of America; Rice University, UNITED STATES

## Abstract

The dynamic signal encoding paradigm suggests that information flows from the extracellular environment into specific signaling patterns (encoding) that are then read by downstream effectors to control cellular behavior. Previous work empirically quantified the information content of dynamic signaling patterns. However, whether this information can be faithfully transmitted to the gene expression level is unclear. Here we used NFkB signaling as a model to understand the accuracy of information transmission from signaling dynamics into gene expression. Using a detailed mathematical model, we simulated realistic NFkB signaling patterns with different degrees of variability. The NFkB patterns were used as an input to a simple gene expression model. Analysis of information transmission between ligand and NFkB and ligand and gene expression allows us to determine information loss in transmission between receptors to dynamic signaling patterns and between signaling dynamics to gene expression. Information loss could occur due to biochemical noise or due to a lack of specificity. We found that noise-free gene expression has very little information loss suggesting that gene expression can preserve specificity in NFkB patterns. As expected, the addition of noise to the gene expression model results in information loss. Interestingly, this effect can be mitigated by a specific choice of parameters that can substantially reduce information loss due to biochemical noise during gene expression. Overall our results show that the cellular capacity for information transmission from dynamic signaling patterns to gene expression can be high enough to preserve ligand specificity and thereby the accuracy of cellular response to environmental cues.

## Author summary

The fidelity of signal transduction depends on the accurate encoding of ligand information in specific signaling patterns and the reliable transmission of these patterns by downstream gene expression machinery. We present an analysis of the accuracy of information transmission from signaling dynamics into gene expression in the case of the transcription factor NFkB. We show that noiseless gene expression can preserve ligand identity with minimal information loss. The addition of noise to gene expression model results in information loss, an effect that can be largely mitigated by choice of parameter values.

## Introduction

The ability of cells to respond to environmental changes is key to their function. A ligand binding to a receptor initiates a cascade of biochemical transformations of cellular kinases, phosphatase, and other enzymes that connect the receptor to downstream effectors, often to change gene expression patterns [1]. Historically, these cascades of events were divided into distinct pathways. The pathway paradigm was appealing because it was easy to understand how information about ligand identity and abundance is preserved. However, as more and more signaling interactions were discovered, it became clear that the linear signaling pathway paradigm is insufficient in describing the complexity of how information propagates between receptors and effectors. The complexity of signaling networks with a large degree of crosstalk and feedback shifted the paradigm from pathway to network-centric view [2]. However, unlike an isolated signaling cascade, the network view poses a challenge. If the mapping between ligands and signaling nodes is many to many, how does specificity, i.e. information about ligand identify and concentration, is preserved?

A plausible solution for the specificity challenge in signaling networks is based on the concept of dynamic signal encoding [3–8]. Information about extracellular events undergoes multiple transformations. Initially, information on ligand identity and abundance is transformed, i.e. encoded, into a specific signaling activity pattern. Subsequently, downstream effectors such as transcription factors transform the specific dynamic signaling patterns to a specific cellular response [9–12]. The dynamic signal encoding view is useful since it explains how information can be preserved despite the complex many to many relationships between receptors and signaling nodes [13]. However, given the current understanding of transcription factor activity, it is unclear whether their dynamics can fully map distinct ligands to different patterns of gene expression. In other words, is the information transmission capacity between signaling dynamics to gene expression sufficiently large to transmit information encoded by these dynamic signaling patterns? It is possible that not all the information that exists within dynamic signaling patterns will be accessible to downstream gene expression machinery [14–16]. Information transmission into gene expression could be limited by specific biochemical constraints of gene expression machinery, i.e. are a property of the gene expression model [17–22] or simply due to additional layer of biochemical noise during the process of gene expression itself [15,23,24].

Information theory can be used to assess the quality of any input/output relationships between two random variables and was used extensively to quantify and understand information transmission in neurons [25–27] and developmental biology [28–31]. As the fundamental function of signaling networks is to reliably transmit specific information about ligand concentration to downstream effectors, information theoretical tools have been utilized as a way to assess the performance of a network [19,20,32–40]. Existing biochemical variability that occurs at multiple timescales can have an adverse effect on the quality of information transmission [38,41–46]. Using information theoretical tools, one can probe the operational quality of a signaling network in a quantitative manner by measuring the information transmission capacity of a network through a series of input/output measurements. Mutual information analysis can quantify the degree of overlap between cellular responses to multiple distinct inputs and thereby it is a good descriptor of the accuracy of information transmission through a signaling network. These tools have been applied to signaling networks showing that indeed information loss occurs due to biochemical noise during the encoding step [16,47–53].

Within the dynamic signal encoding paradigm, information transmission depends on the information that exists within the dynamic signal as well as the ability to transmit this information into specific gene expression patterns. If gene expression is noisy and inaccurate, a high-quality encoding is of little use to the cell. Therefore, the question of how accurate is gene expression in capturing information from dynamic signals is paramount to our understanding of the overall performance characteristics of signaling networks. Previous work addressed this question using experimental and using theoretical approaches. Experimental measurement of the accuracy of mapping between signaling dynamics to single-cell gene expression is technically challenging. Work by Hansen et al [16] analyzed the degree of gene expression accuracy with regards to oscillatory signal and amplitude. While useful, it is unknown if cells rely on amplitude and frequency as the key features. In a more physiological setting, Lane et al measured the correspondence in a single cell between NFkB dynamics and resulting gene expression [54]. They were able to demonstrate that indeed the overall patterns of signaling dynamics result in distinct expression patterns. However, a high level of measurement error and a small sample size preclude an analysis of the reliability of information transmission accuracy from signaling dynamics into gene expression processes. Theoretical work on this question was more extensive [19,55–58]. However past work based the analysis on specific dynamic features (e.g. frequency or amplitude) or used simplified input signals that are unrealistic. Therefore, there is a gap in our understanding of the degree of accuracy of information transmission from ligand to gene expression patterns for realistic signaling dynamics patterns.

Here we address this gap by analyzing the information transmission accuracy of a simple gene expression model. We utilize NFkB signaling, as it is a system where dynamic signal encoding has been analyzed in depth [47–49,59–62]. We first generate simulated encoding data for multiple ligands that activate NFkB and ask how accurate is the mapping of this dynamic into distinct gene expression patterns. We show that without noise, gene expression accuracy is close to its maximal possible value for a large range of parameter values. However, when noise in gene expression is considered performance decreases. Still, close to maximal information transmission can still be achieved by a specific choice of gene expression model parameters. Overall our finding demonstrates that information transmission from signaling dynamic into gene expression can be achieved to allow cells to accurately respond to environmental changes.

## Results

**Fig 1:**
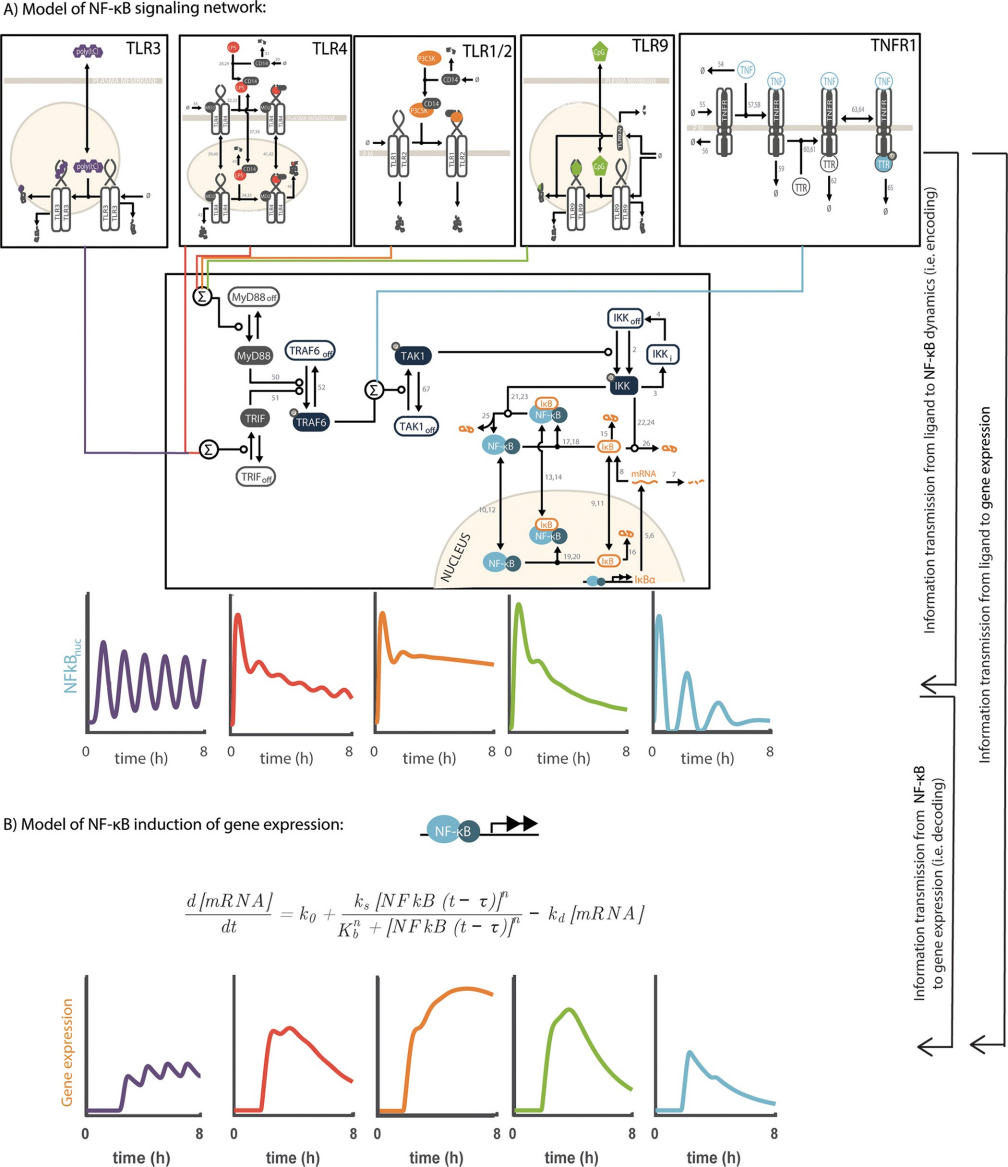
Models of dynamic signal encoding and gene expression in the NFkB network. **A**. The encoding model of NFkB signaling network. The model includes five distinct receptor modules (TLR3, TLR4, TLR1/2, TLR8, TNFR1) that feed at three distinct points (MyD88, TRIF, and TAK1) into the core IKK/NFkB module. The dynamic interaction in network results from the mapping between each receptor module and dynamic activation profiles of the transcription factor NFkB. **B**. The gene expression model contains a simplified a single ordinary differential equation that uses the nuclear concentration of NFkB over time as an input and produces the gene expression pattern as an output. The model effectively maps the dynamic of NFkB into dynamic gene expression profile.

We first constructed mathematical models that represent the dynamic signal encoding and information transmission into gene expression steps in NFkB signaling (Fig 1). The first model (Fig 1A) captures the encoding step and the transformation of ligand identify to the dynamics of NFkB. The second model (Fig 1B) focuses on gene expression based on NFkB dynamics. As the goal of the encoding model was to capture the specific aspects of NFkB dynamics we opted to use a highly detailed model with 95 reactions of 48 reactants and 127 parameters [63]. The model connects five different receptors: TNFR, TLR4, TLR3, TLR9, and TLR1/2 through the key downstream pathways of MyD88 and TRIF to the IKK modules that controls NFkB dynamics [64]. The model generates realistic simulated time series up to 8 hours that match experimental data for five different ligands [65]. Given the scope of the model and the fact that includes multiple inputs to the same core network we focused on the question of ligand specificity and tested one ligand concentration for each condition (i.e., TNF = 10ng/ml, LPS = 10ng/ml, PolyIC = 30μg/ml, CPG = 1μM, Pam3CSK = 300ng/ml). The ligand concentrations were chosen to be close to the corresponding receptor Kd [63]. The construction of these two models is based on a functional separation of encoding ligand identity into NFkB dynamics and the transmission of this information into gene expression patterns. This functional separation is not the only one as the NFkB network by itself includes gene expression. However, this separation is useful in addressing the question of information transmission from NFkB dynamics into gene expression. Furthermore, this separation makes needed simplification that only NFkB dynamics matter while it is well known that these ligands not only regulate differential NFkB profiles but also activate additional transcription factors such as interferon regulatory factors (IRFs), kinases of the c-Jun N-terminal kinase (JNK) and MAPK/ERK families [66]. The key benefit of using a mathematical model to capture NFkB dynamic profile is that it allows us to control the degree of encoding noise, a manipulation that is technically impossible to perform experimentally. Furthermore, the analysis avoids the need to deal with experimental and technical measurement errors. The second model (Fig 1B) captures the gene expression step. Unlike the encoding model that aims at a highly realistic details description of the underlying system, the gene expression model is very simple with a single differential equation and six parameters. We chose a simple model to allow us to capture the essence of the gene expression step without unnecessarily increasing the number of parameters. Using the two models in tandem we can simulate how cells respond to extracellular stimuli through both NFkB signaling dynamics and resulting gene expression at multiple degrees of biological extrinsic noise, as was shown to be important for information capacity of signaling dynamics [44,67].

**Fig 2:**
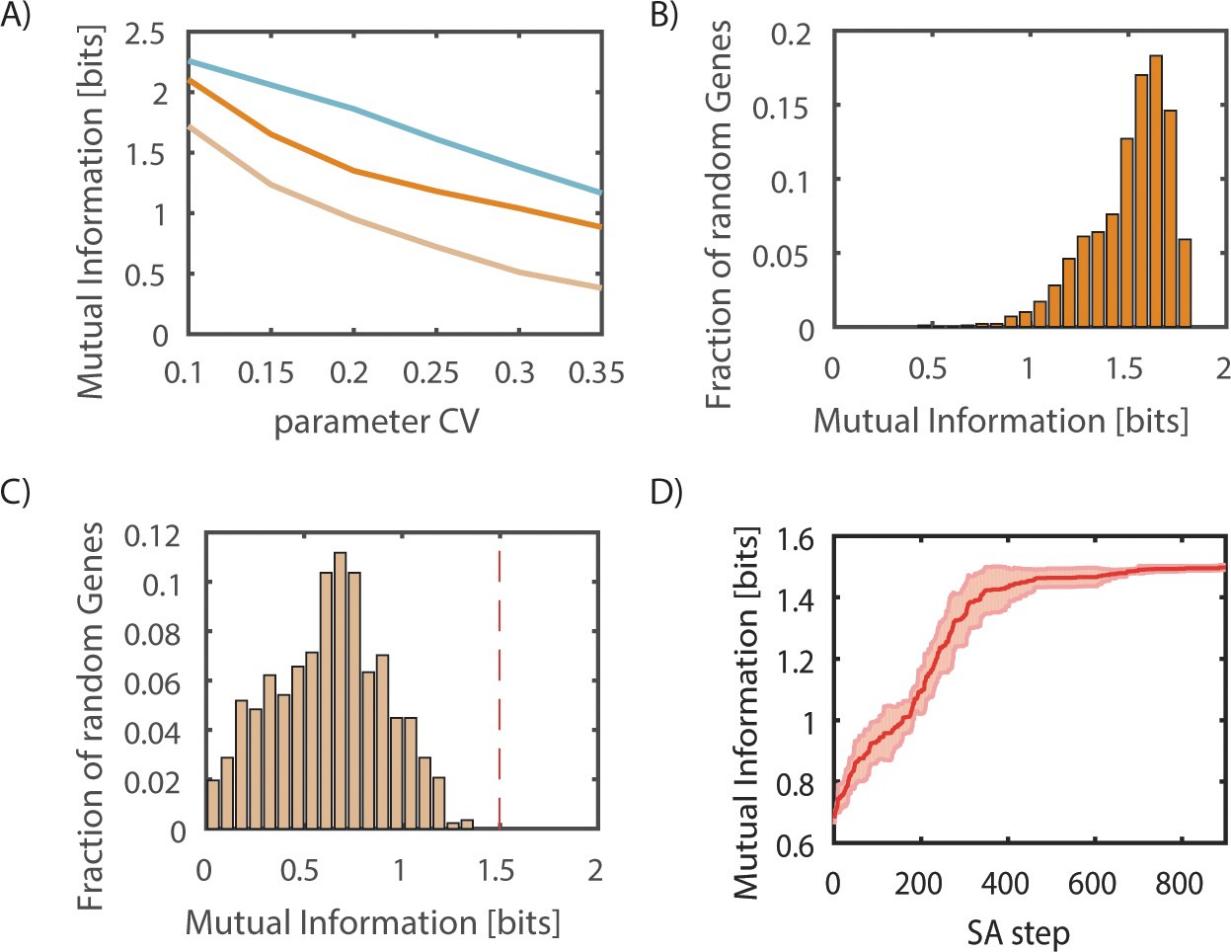
Information loss in dynamic signal encoding and gene expression. **A.** Mutual information as a function of parameter variability that exists in the encoded in NFkB dynamics (cyan), and noiseless (dark orange) and noisy (light orange) information transmission of NFkB dynamics into simple gene expression model. Values are the mean of 1000 samples. **B** and **C** distribution of mutual information values in noiseless (**B**) and noisy (**C**) information transmission of NFkB dynamics into a simple gene expression model. The distribution comes from the inference of mutual information after sampling different model parameters. In each case, 1000 parameter values are sampled from a log-normal distribution centered around reference values with a CV of 25%. The mutual information achieved after optimization of the parameter values of the gene expression model, the mean of panel B MI = 1.5, is shown as a red dashed line in panel C. **D**. Optimization of parameter values using heuristic simulated annealing show convergence of mutual information values. The red line shows the average and shading the standard deviation from 4 repeated optimization runs.

To analyze the effect of cellular heterogeneity on cellular information transmission we estimated the effect of biochemical variability on the accuracy of signal transduction using an information theoretic approach. The dominant source of variability in many signaling networks [41,47,68] and in NFkB specifically [69] is differences between cells in their underlying cell state (e.g. protein concentration, organelle structure, etc) between cells. To capture this effect, we repeated the simulation of the encoding model 1000 times per condition with parameter drawn from a log-normal distribution centered around the reference value with different degrees of the coefficient of variation. Our model makes the simplifying assumption that stochastic effects during dynamic signal encoding (i.e. intrinsic noise) are of smaller magnitude. We tested a range of variability magnitudes from 10–35% and at each condition estimated the mutual information between ligand identity and NFkB dynamics (Fig 2A, cyan line). As expected, the accuracy of the encoding step depended on the degree of biochemical variability. At low values (10%) there was very little information loss compared to the maximum possible value of log_2_(5) ~ 2.3. As expected, as the magnitude of variability increased the signaling accuracy decreased [44].

Next, we used the simulated trajectories as input to the gene expression model and asked how much additional information loss will occur during information transmission into gene expression. To separate the effect of biochemical variability during signaling and gene expression we first tested a gene expression model without biochemical variability. To make the analysis independent of the specific parameter values of encoding we sampled 1000 different parameter values of the 127-dimension parameter vector. We found that the noise-free gene expression model is able to capture most of the information that exists within NFkB dynamics (Fig 2A and 2B and Table B in the S1 Text). This indicates that the five tested ligands generate sufficiently distinct NFkB activation profiles such that a simple model of gene expression is sufficient to generate distinct gene expression profiles that provide sufficient information capture the ligand identity to a similar degree that it exists in NFkB dynamics.

The analysis above takes into account noise within the encoding step but ignores the existence of noise within the gene expression step. As it is likely that biochemical variability plays a role in gene expression we tested the overall information transmission fidelity in the presence of noise in gene expression. The addition of biochemical variability into the gene expression step resulted in additional loss of information. This was true for large ranges of parameter values for the gene expression model (Fig 2C).

To maximize signaling fidelity, information loss should be minimized. We tested whether the information loss we observe during the gene expression step is an unavoidable property of the system, or whether it is possible to achieve higher fidelity information transmission with optimized parameters. We performed heuristic optimization of the gene expression model parameters with the objective function of maximizing the mutual information between ligands and gene expression dynamics (Fig 2D). In the figure, line and shading represent mean and standard deviation, respectively, generated from 4 repeated optimization runs of gene expression parameters (S1 Text). Interestingly, with the right parameters, there was little loss of information during gene expression step (Fig 2C red dashed line) despite the existence of biochemical noise in the gene expression model. Repeating the optimization multiple times identified similar parameter values. Overall these results indicate that careful choice of the population average gene expression parameters can mitigate the effect of biochemical variability around these reference values (Table A in the S1 Text). These results suggest that cells can potentially adopt an information transmission strategy to maximize the information extraction from the signal encoded by NFkB dynamics.

We next wanted to better understand the identified strategy that maximizes information transmission by answering two questions related to the identified solutions: 1. What changes in gene expression patterns cause it to increase its information about ligand identity and 2. What model parameters are changed and do these changes contribute to the increase in information transmission accuracy?

**Fig 3:**
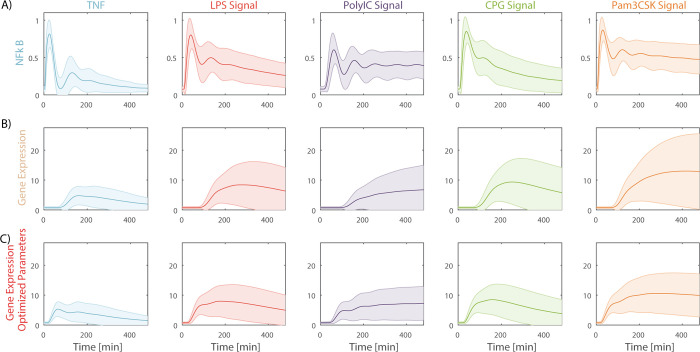
Simulation of NfkB dynamics and resulting gene expression. Average (center line) and standard deviation (shading) of NFkB dynamic (**A**), noisy gene expression model (**B**) and noisy gene expression model centered around parameter values that allowed maximal information transmission (**C**). Each condition was simulated 1000 times with parameters of both signaling and gene expression models sampled from a log-normal distribution with a CV of 25%.

**Fig 4:**
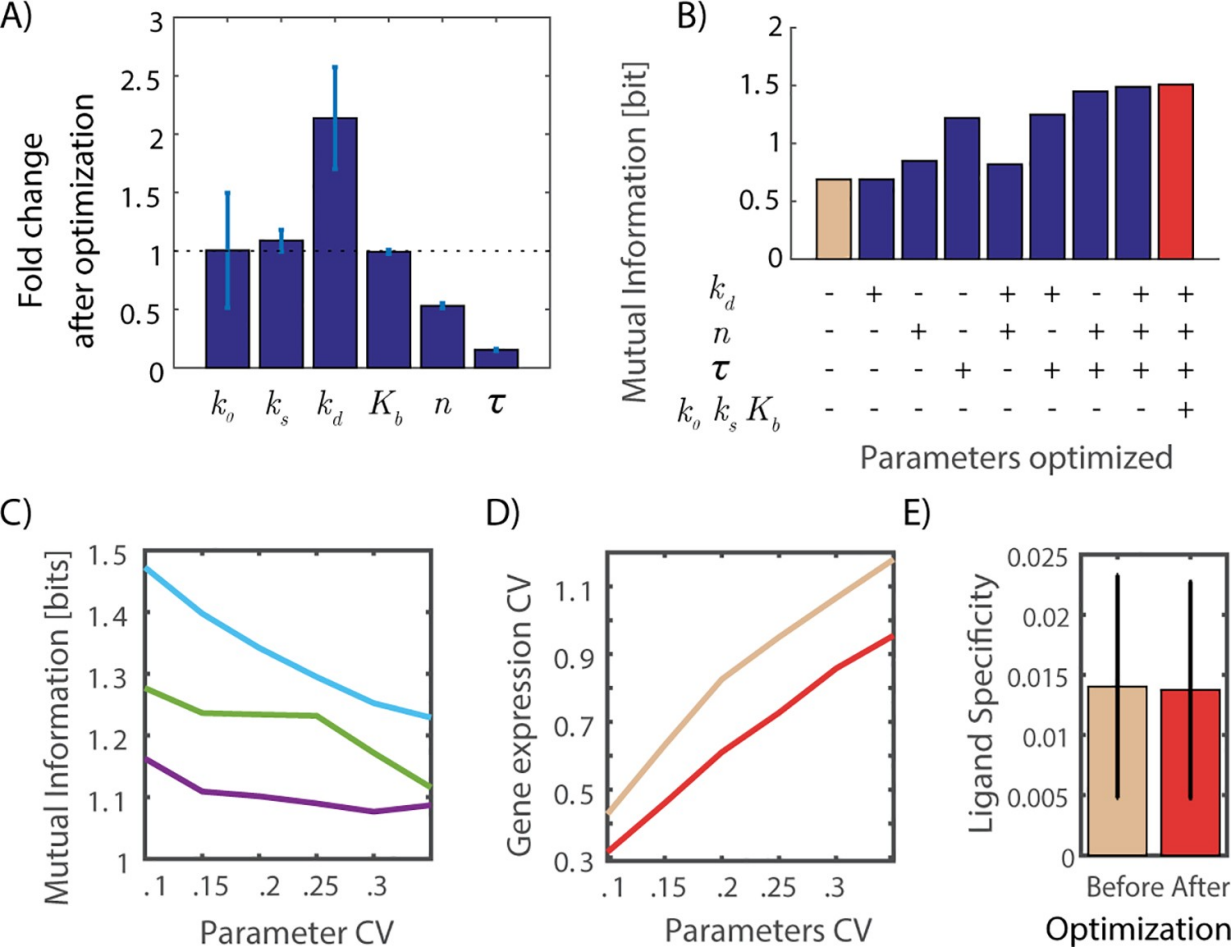
Effects of mutual information optimization on gene expression. **A.** Changes in the six parameters of the gene expression model as a result of optimization to increase mutual information. Error bars show standard deviation from 4 repeated optimization runs. **B.** Mutual information with all parameters used at nominal values (light orange), optimized values (red), or a mixture of nominal and optimized values (blue bars). **C.** Mutual information as a function of the amount of variability in a single model parameter shown for mRNA degradation rate k_d_ (purple), hill coefficient n (green) and time delay *τ* (cyan). All parameters considered at nominal values. **D**. Change in gene expression variability with an increase in signaling and gene expression models parameter variability for nominal (light orange) and parameter optimized (red) gene expression models. **E** Ligand specificity was calculated as the mean square error between the average responses to the five ligands shown before and after parameter optimization to maximize mutual information.

To assess what changed about the system after optimization we examined the dynamic trajectories of NFkB and resulting gene expression patterns (Fig 3). A strength of mutual information analysis is that it incorporates both differences in the overall separation between average responses to ligands and the variability within a response to a single ligand into the same framework. Analysis of the gene expression pattern before and after optimization indicates that the major change in gene response after optimization is a reduction in response variability per each ligand (Fig 4D) and not from an increased separation of the typical response to all ligands (Fig 4E). Interesting, mutual information was insensitive to up to 2-fold changes in dose of LPS or TNF suggesting that the ability to discriminate between ligands is not dose specific (Figure C and Figure D in S1 Text).

Next, we examined the contribution of each of the identified parameters to the increase in information transmission accuracy. Of the system’s six parameters, only three changed their value in a consistent manner (Fig 4A): mRNA degradation rate (*k*_*d*_), the cooperativity of the binding reaction (n), and the time lag between increased NFkB in the nucleus to changes in expression (*τ*). The bar graph values of each parameter, mean and standard deviation were calculated from the optimized parameter distribution that is generated from 4 repeated SA runs. Figure E in the S1 Text also depicted the distribution of optimized gene expression parameters. However, the fact that a parameter changed through our optimization does not necessarily guarantee that this change is meaningful and is responsible for the increase in mutual information. To assess the specific contribution of these three parameters we score the information transmission accuracy for all possible combinations of these parameters for a specific value of variability (25% parameter CV). In each test, all other parameters have their original reference value before optimization. We saw that the parameter that contributed most to the reduced variability is the time delay parameter tau (Fig 4B). This was true for a wide range of degree of biochemical variability (Fig 4C). Given the dynamic nature of NFkB, a reduction in the value (and variance) of the time delay unifies the population response and can explain the reduction in gene expression variability we observed (Fig 4D). Interestingly, the lower value (14.22 minutes) matches the experimental estimate of this delay for genes that are part of the NFkB network such as A20 and IkB [62].

## Discussion

Metazoan signaling architecture is unique with a high degree of crosstalk through the network [70]. The benefits of this architecture are that it is more plastic and allows multicellular organisms to have specialized cells type that responds differently to the same environment without exponentially growing number of pathways [69]. However, this benefit comes at a cost. Cells need to accurately encode stimulus information in specific signaling patterns that can be transmitted to downstream effectors. Biochemical variability in either dynamic signal encoding or gene expression will prevent the accurate response to changes in the extracellular environment.

In this study, we analyzed the fidelity of information transmission from ligands, through signaling dynamics into gene expression patterns. We generated realistic signaling patterns using a complex mathematical model and asked how much information about ligand identity is lost in the transmission of signaling dynamics into gene expression patterns. It is important to emphasize that the information transmission of signaling dynamics into gene expression patterns does not necessarily imply perfect classification of dynamic patterns into completely distinct patterns. It is possible that the response to different dynamic patterns of transcription factor activity will result in overlapping expression patterns. Indeed, the overall information transmission between ligands and gene expression indicates that information was lost during transmission. Our results indicate that for realistic patterns of NFkB dynamic information transmission into gene expression is capable of preserving most of the information that is encoded in the signaling patterns of NFkB and that most of the information loss occurs during dynamic signal encoding.

We focused on information transmission into gene expression based on a simplified model with a single transcription factor and a single gene. This is a simplification as it was shown that the collective dynamics of multiple transcription factors are needed to encode sufficient information on the extracellular environment to generate signal specific downstream responses [51]. It is interesting to ask whether more complex gene expression models, i.e. multiple genes or more complex models of a single gene that were shown to be important for NFkB [60], can further increase information transmission fidelity. Our results show that even a single gene can recover most of the information that exists within NFkB dynamics. Therefore, while additional genes or more complex models of expression from a single gene that allow promoter to transition between states or include feedforward regulation will undoubtedly have higher capacity, information transmission will be bounded by the information loss in the encoding step itself. Where more complex gene expression models might be important is in allowing model parameters that are more restrictive. Restrictive parameters could be a result of evolutionary constraints and the need for different cell types to show distinct responses to the same ligands. Under such conditions, a more complex gene expression model might increase the overall information transmission fidelity. Similarly, more complex encoding, i.e. capturing ligand identify through the dynamics by multiple transcription factors, can only improve overall information transmission fidelity. Furthermore, our analysis was based on mapping the entire dynamics (i.e. full timeseries) of NFkB into the dynamics of gene expression. It will be interesting to analyze what aspects of the dynamic patterns are more meaningful using either a features based approach [56] or through the use of information transmission rate [57].

The numerical analysis we performed identified specific values that can be used to maximize transmission from signaling dynamics into gene expression patterns. It is interesting to ask whether 1) these parameters are physiological, and 2) what is the intuition behind the values generated by the optimization procedure. Given the simplicity of the model, it is unlikely that all genes in a cell have these parameters. It is likely that the simplistic model used here truly captures the complexity of physiological gene expression responses and therefore the physiological relevance of the specific parameter values identified is limited. However, that does not mean that the parameter values themselves do not have any meaning, intuition developed by interpreting these values could have significance to larger more complex models. Our optimization showed that the key parameters changes were RNA half-life, the delay between signal and gene expression and cooperativity in gene expression. The effect of lowering RNA half-life is that more of the high frequency information in the dynamic patterns is preserved, pointing out that indeed fast changes in NFkB dynamics on the order of ~20 minutes are meaningful. The reduction in the delay is a bit more nuanced. In a deterministic system the delay value would simply cause a shift between signaling and expression and should not cause any changes to mutual information. However, with the addition of parameter variability a lower mean delay value, that will have lower variance, will help unify the mapping between NFkB and gene expression. Finally, the reduction in cooperativity could be explained as an effective increase in the dynamic range of the dynamics to expression mapping. Overall, while the specific values of the identified parameters should not be taken at face value, it is likely that the principles exposed by these changes will hold in more complex physiological setting.

Dynamical signaling patterns are observed in multiple signaling systems. Our analysis focused on a single case study of NFkB. It will be important to address questions of information transmission fidelity, both computationally and experimentally, in other signaling systems. As our understanding of the role of signaling patterns increases so is the desire to utilize this understanding to design better therapeutic approaches [71,72]. Understanding the mechanism of accurate information transmission from dynamic signals into gene expression, and the constraints that influence information transmission fidelity is an important step in the rational design and manipulation of these dynamical targets [71].

## Methods

### Quantification of information transduction

To estimate mutual information transduced by NFkB and downstream gene, we applied a binless strategy [47]. This approach uses an embedding of each simulated temporal response into a vector space and calculates Shannon’s entropy based on the k-nearest neighbor Euclidean distance within these vector spaces. We started derivation of an algorithm to quantify information flow by considering a 5-dimensional column input signal vector *L*, where each vector element defines corresponding extracellular ligand concentration (*L* = [*l*_1_,*l*_2_,*l*_3_,*l*_4_,*l*_5_]). After exposure to each *l*_*i*_ ligand, cells response (time trajectories of NFkB and downstream gene: $R_{i}=[r_{i1},r_{i2},\ldots \ldots ..,r_{in_{i}}]$) differentially due to cell-to-cell parameter variability. Each response trajectory was projected as a single point in continuous Euclidean space of dimension *d*, where *d* is the number of time points picked from an output trajectory. From log-normal distribution containing reference mean and CV values of each parameter, we sampled 1000 parameter sets for *in silco* experiment under each signaling condition. Thus, we generated $N=\sum_{i=1}^{5}n_{i}=5000$ response trajectories in our response *R* = [*R*_1_,*R*_2_,*R*_3_,*R*_4_,*R*_5_] array that mitigates to construct a response distribution in *d* dimensional space having 5000 points. Following published formalism [66,73] accomplished with *k*-nearest neighbor estimator, we quantified Shannon differential entropies such as the sum of the conditional entropy of each signal to get overall conditional entropy and non-conditionall entropy *H*(*R*). Both entropies quantification are unattainable without the knowledge of input signals’ (*L*) probability (*q*_*i*_). However the maximum information transduction defined as channel capacity is calculated by maximizing function in optimization method to optimize *q*_*i*_’s such that $\sum_{i=1}^{5}q_{i}=1$ where *q*_*i*_≥0. In this study, we considered equiprobable ligands probability ($q_{i}=\frac{1}{5}$) because our observable is mutual information not channel capacity. Using the classical formula (*MI*(*R*;*L*) = *H*(*R*)−*H*(*R*|*L*)), information flow (*MI*) between input (*L*) and output (*R*) was estimated. In encoding step *MI* calculation, *R* array was generated from NFkB trajectories whereas downstream gene trajectories gave *R* array in gene expression step *MI* calculation

To convert a dynamic response into a *d* dimensional vector form, we have to opt for the appropriate time points. A response (*R*) array, evaluated at the time points, gives maximum information tranduction. We applied a simple strategy to determine vector dimension and the time points. We equally spliced the time frame of response dynamics (1 to 480 minutes) into *V*+2 number to get dimension *V*, after removing the first and last value. For *V* = 1, it choses response value at the center of the time frame and *V* = 2, it picks values at one third and two thirds of the time frame. In our analysis, we calculated *MI* for encoding (NFkB) and gene expression dynamics up to *V* = 8 (Figure A in S1 Text) considering 25% parameter CV. The results indicate that *MI* value converge. To avoid the curse of dimensionality in *MI* calculation, we considered response value at five [50, 100, 150, 300, 450] time points (in minutes) not only to generate vector space that capturing maximum dynamical feature of NFkB and target gene but also to attain the converged *MI* values. In *MI* estimation, we used *k* = 10 for k-nearest neighbor Euclidean distance calculation.

We checked possible bias in sample size associated with *MI* value that asymptotically converges for a large population of cell (sample size→∞). *MI* vs inverse of sample size measurement, performed at aforementioned time points, shows nearly horizontal line for encoding and gene expression response (Figure B in S1 Text). The results indicate the bias in mutual information estimation is nominal for our considering sample size.

### Stochastic optimization

Optimization was implemented using simulated annealing (SA) to decipher the gene expression model parameter set that can transduce maximum information (Maximal transmission Parameter Set = *arg max MI*(*Gene*[*parameters*])). The optimization objective function follows an information-maximization approach between ligand and gene expression. During the simulation, a randomly chosen variable among 6 gene regulation variables was allowed to make a move for each sampling. The maximum step length taken in our simulation is of 5% with respect to the value of the particular variable (in log10 scale) at the prior sampling step. To be explicit, for any parameter (Param), the update was done by the SA rule, Param0 = Param + Param × (−1)^n^× δ × rand, where Param0 is the updated value of Param, n is a random integer [0 or 1], δ is the amplitude of allowed change (kept 5%), and rand is a uniform random number between 0 and 1. Using the information of the updated parameter, mutual information is calculated for the new set of vector spaces after each iteration and rejection or acceptance of updated parameter obeys SA rule. With the progress of iteration, the mutual information initially goes up and converges as the output becomes close to the desired value. The mutual information will be maximal if MI value at the i^th^ step of the iteration is close to the i-1^th^ step

$$\Delta MI={MI}_{i}-{MI}_{i-1}\approx 0$$

In the optimization code, the precision criterion to terminate the iteration of SA running is *ΔMI*<10^−2^. During the optimization, we didn’t impose any boundary constraints on parameters value. Optimized parameter sets that are very close to each other, however each optimization run was started from different guess parameter sets. At this point, it is important to mention that we didn’t investigate how parameters in the encoding step change information transmission from ligand to dynamic signaling patterns. The encoding model contains 95 kinetic parameters that are quite difficult to optimize.

### Parameter sampling

To simulate with extrinsic noise, model parameters were sampled for the dynamic signal encoding and gene expression models (Fig 1A and 1B). For the encoding models, parameters were sampled from a log-normal distribution centered around the reference values with an identical coefficient of variability (CV) for all parameters. For the gene expression model, parameters were also sampled from a log-normal distribution centered around the guess or optimized values with an equal coefficient of variability (CV) for all parameters. To find good guess or starting parameters value for optimization, we used a multivariate 6-dimensional uniform distribution. Distribution scales are bounded within biologically relevant parameter range i.e., *k*_0_ = [27*e*^−4^ 0.7], *k*_*s*_ = [6*e*^−2^ 1.72], *k*_*d*_ = [5*e*^−4^ 23*e*^−3^], *k*_*b*_ = [4*e*^−2^ 2.46], *n* = [2,6], *τ* = [24 120].

Here all parameters are unit less expect *τ* in minutes. Thus, we divided all NFkB response by the maximum response of it among the 5 ligands condition to make it dimensionless that regulates a non-dimensionalized ODE model of downstream gene. We sampled encoding parameters from a log-normal distribution centered around reference values with CV of (10–35)% and calculated *MI* for the encoding step (Fig 2A).

### Ligand specificity calculation

To address ligand specificity in downstream gene expression, we evaluated population average response of gene expression for all time points in pre and post optimized parameters conditions for each ligand (mean of 1000 trajectories) and for all ligands (mean of 5000 trajectories). We scaled all the average responses by the total value (sum of all time points) of the average response of all ligands and quantified Euclidean distance between the response of each ligand and all ligands. Fig 4E depicts the mean and standard deviation of 5 Euclidean distances associated with 5 ligands.

## Supporting information

S1 Text Supporting text related to methods used in this paper. The supplementary text includes five figures and a table that support the validity of some of the technical aspects of the methods used.
(PDF)
