# Supplementary material for: Information transmission from NFkB signaling dynamics to gene expression
Source: PLoS Comput Biol. 2020 Aug 14;16(8):e1008011. doi: 10.1371/journal.pcbi.1008011 (PMC7478807; doi:10.1371/journal.pcbi.1008011)
Supplement: S1 Text — The supplementary text includes five figures and a table that support the validity of some of the technical aspects of the methods used. (PDF) [file pcbi.1008011.s001.pdf]

Optimal information transmission of NFkB signaling dynamics

Alok Maity[1], Roy Wollman[1,2,3]

1. Institute for Quantitative and Computational Biosciences, University of California, Los Angeles.
2. Departments of Integrative Biology and Physiology and Chemistry and Biochemistry, University of California UCLA.
3. Corresponding author: rwollman@ucla.edu

Table A: Parameter set of the gene expression model from an optimization (SA) run.

Optimization	Basal synthesis rate const. (k_0)	mRNA synthesis rate const. (k_s)	mRNA decay rate const. (k_d)	Binding rate const. (K_b)	Hill coefficient (n)	Time-lag (min) (τ)	**MI**(bits) of Gene expression model
Before	5e-3	0.3	7e-3	0.72	6	93.7	0.69
After	7e-3	0.3	11e-3	0.71	3.4	14.1	1.49

Table B: Mutual information in encoding step (Considering 25% parameter CV) and gene expression step in noiseless and noisy (Considering 25% parameter CV) conditions.

Observable	NFkB (encoding step)	Random Gene (noiseless)	Random Gene (noisy)
**MI** (bits)	1.61	1.18 (73.29%)	0.72 (44.72%)

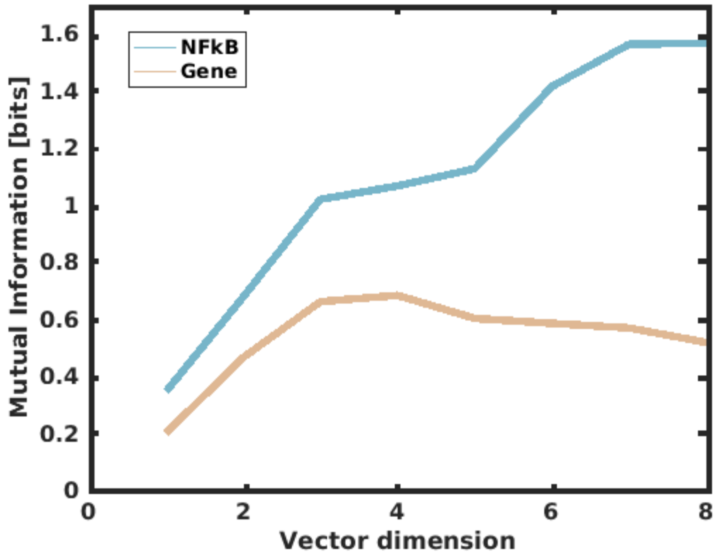

Figure A: Information transduction as a function of the dimension of multivariate dynamical responses. Multivariate response vector is subsampled from the equally spliced time frame centered on middle time point of response dynamics. Mutual information of decoding (cyan) model and gene (light orange) expression model (noisy condition) with gene expression parameters at nominal values. In both cases, 25% parameter CV is considered.

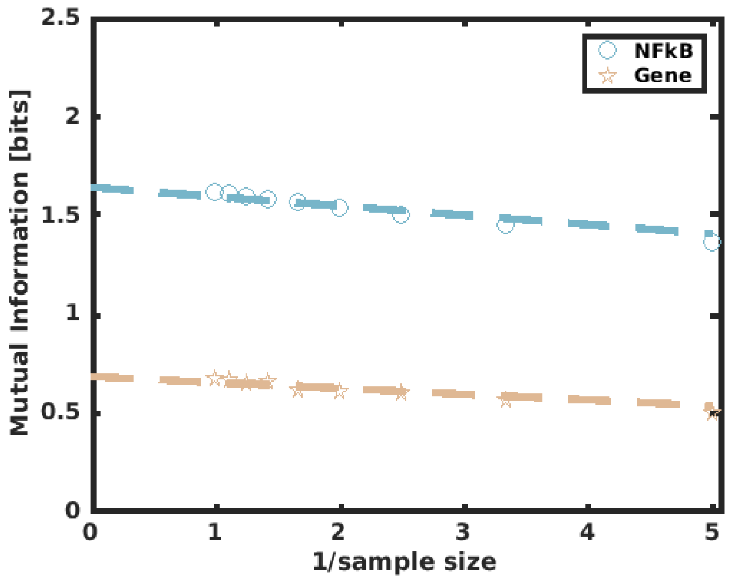

Figure B: Sampling bias in mutual information estimation. Mutual information (MI) as a function of inverse of sample size (no. of response trajectories) of decoding (cyan) model and gene (light orange) expression model (noisy condition) with parameters at nominal values. Points represent computed MI value and broken lines are obtained from a linear fitting of the points. The corresponding y-intercept characterizes the information transfer for infinite sample size.

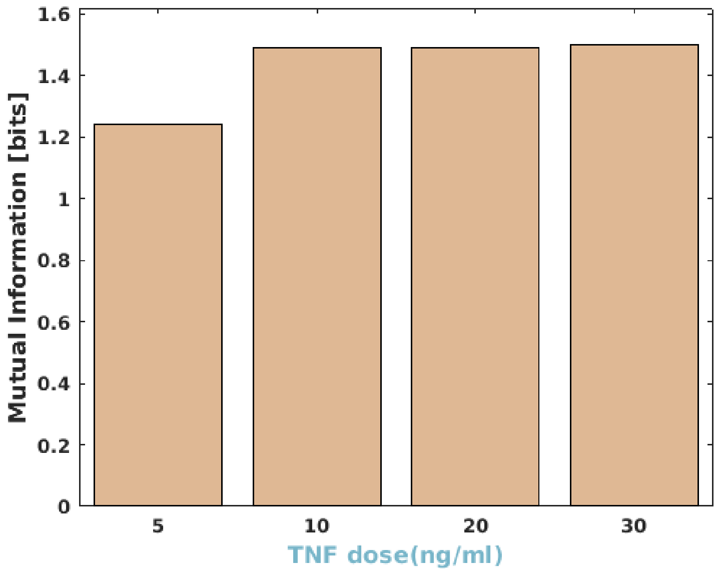

Figure C: Specificity of TNF ligand-dependent gene response. Quantification of mutual information of gene expression model for different doses of TNF keeping doses of the remaining ligands fixed that mentioned in the results section. Calculation is conducted using the optimized gene expression parameters with 25% CV of log-normal parameter distribution.

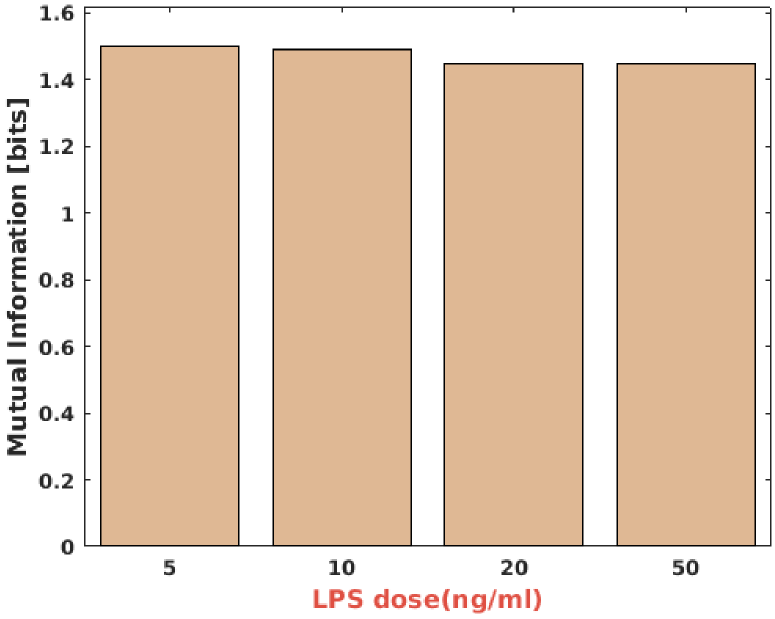

Figure D: Specificity of LPS ligand-dependent gene response. Quantification of mutual information of gene expression model for different doses of LPS keeping doses of the remaining ligands fixed that mentioned in the results section. Calculation is conducted using the optimized gene expression parameters with 25% CV of log-normal parameter distribution.

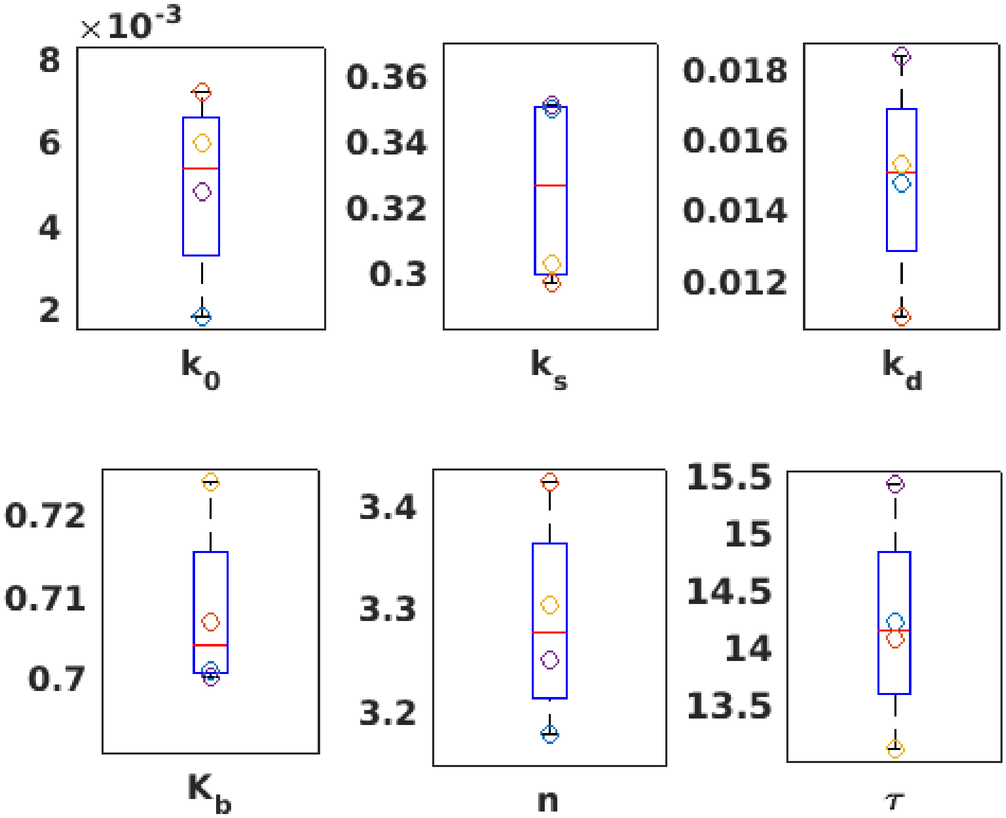

Figure E: Boxplot of optimized gene expression parameters. 4 repeated optimization (SA) of gene expression model parameters with the objective function of maximizing mutual information is conducted. Each point represents outcome of each optimization run.
